# Integrated application of transcriptomics and metabolomics provides insights into glycogen content regulation in the Pacific oyster *Crassostrea gigas*

**DOI:** 10.1186/s12864-017-4069-8

**Published:** 2017-09-11

**Authors:** Busu Li, Kai Song, Jie Meng, Li Li, Guofan Zhang

**Affiliations:** 1 Key Laboratory of Experimental Marine Biology, Institute of Oceanology, Chinese Academy of Sciences, Qingdao, China; 2 University of Chinese Academy of Sciences, Beijing, China; 3 Laboratory for Marine Biology and Biotechnology, Qingdao National Laboratory for Marine Science and Technology, Qingdao, China; 4 Laboratory for Marine Fisheries and Aquaculture, Qingdao National Laboratory for Marine Science and Technology, Qingdao, Shandong China; 5 National & Local Joint Engineering Laboratory of Ecological Mariculture, Institute of Oceanology, Chinese Academy of Sciences, Qingdao, China

**Keywords:** Pacific oyster, Glycogen, Amino acid, Free fatty acid, Quality trait

## Abstract

**Background:**

The Pacific oyster *Crassostrea gigas* is an important marine fishery resource, which contains high levels of glycogen that contributes to the flavor and the quality of the oyster. However, little is known about the molecular and chemical mechanisms underlying glycogen content differences in Pacific oysters. Using a homogeneous cultured Pacific oyster family, we explored these regulatory networks at the level of the metabolome and the transcriptome.

**Results:**

Oysters with the highest and lowest natural glycogen content were selected for differential transcriptome and metabolome analysis. We identified 1888 differentially-expressed genes, seventy-five differentially-abundant metabolites, which are part of twenty-seven signaling pathways that were enriched using an integrated analysis of the interaction between the differentially-expressed genes and the differentially-abundant metabolites. Based on these results, we found that a high expression of carnitine O-palmitoyltransferase 2 (CPT2), indicative of increased fatty acid degradation, is associated with a lower glycogen content. Together, a high level of expression of phosphoenolpyruvate carboxykinase (PEPCK), and high levels of glucogenic amino acids likely underlie the increased glycogen production in high-glycogen oysters. In addition, the higher levels of the glycolytic enzymes hexokinase (HK) and pyruvate kinase (PK), as well as of the TCA cycle enzymes malate dehydrogenase (MDH) and pyruvate carboxylase (PYC), imply that there is a concomitant up-regulation of energy metabolism in high-glycogen oysters. High-glycogen oysters also appeared to have an increased ability to cope with stress, since the levels of the antioxidant glutathione peroxidase enzyme 5 (GPX5) gene were also increased.

**Conclusion:**

Our results suggest that amino acids and free fatty acids are closely related to glycogen content in oysters. In addition, oysters with a high glycogen content have a greater energy production capacity and a greater ability to cope with stress. These findings will not only provide insights into the molecular mechanisms underlying oyster quality, but also promote research into the molecular breeding of oysters.

**Electronic supplementary material:**

The online version of this article (doi:10.1186/s12864-017-4069-8) contains supplementary material, which is available to authorized users.

## Background

The Pacific oyster *Crassostrea gigas* is an important marine fishery resource cultivated globally, and the annual global production continues to increase at a rate of four million tons per year (FAO, 2014, http://www.fao.org). As the largest oyster producing country, China produces more than 80% of the total global production. Glycogen makes up 20–40% of the oyster’s dry weight, and as the main flavor component the glycogen content is critical to oyster quality. The glycogen content is not only related to the flavor and the quality of the oyster, but is also related to its hardiness. As a high-efficiency energy storage form, glycogen in oysters is bound up with summer mortality [1] and energy metabolism. A high glycogen content can also promote gonad development and gametogenesis [2].

Glycogen is a branched glucose polymer exist in animals and fungi across numerous taxa [3]. As a critical nutrient, glycogen content had been extensively studied in many domesticated animals including sheep, pigs, rabbits, and chickens [4–7]. Glycogen metabolism is highly conserved across species, with glycogen phosphorylase and glycogen synthase being the key enzymes in glycogen degradation and glycogen synthesis, respectively. Several glycogen metabolism-related genes have also been identified in *C. gigas*. Hata’s group has reported the extraction and purification of glycogen phosphorylase from the adductor muscles of oysters [8], whereas Bacca’s group reported the cloning of the oyster glycogen synthase gene, and that the expression level of glycogen synthase changed in a pattern consistent with seasonal variation in glycogen content [9]. In other mollusks, such as *Crassostrea angulata*, glycogen synthase and its regulator glycogen synthase kinase 3 have been characterized, and it was found that their expression depends on the reproductive cycle [10]. Additionally, several recent studies have described the molecular regulation of enzymes involved in glycogen metabolism in *C. gigas* [11, 12]. In this regard, a quantitative trait loci (QTL) analysis identified two SNPs in the glycogen debranching enzyme and one SNP in glycogen phosphorylase that are associated with glycogen content [11]. A more recent study found an effective haplotype of glycogen synthase is significantly related to the glycogen content in *C. gigas* [12]. Zhong’s group also found two QTLs associated with glycogen content and showed that the left shell depth and volume might be used as an indirectly selected indicator of glycogen content [13]. In pig skeletal muscle, based on the integration of QTL and GWAS (Genome Wide Association Study), a splice mutation in the PHKG1 gene was found that results in a high glycogen content [14, 15]. However, in oysters, little is known about the genetic mechanisms that determine glycogen content and therefore, a significant amount of work remains to be done.

Since carbohydrates such as glycogen, amino acids, and fatty acids are the three major nutrients in organisms, their metabolic pathways are interconnected. Increased levels of free fatty acids may lead to an increase in glycogen content by reducing glycogen consumption [16]. Similarly, a high protein diet could increase the glycogen content because a significant portion of the dietary amino acid is channeled towards glycogen synthesis [17]. However, the molecular mechanisms and the genes that mediate these connections in oysters have not been discovered and require an in-depth study.

Over the past decade, rapid advances in technology have made next-generation sequencing suitable to conduct large-scale searches to identify candidate genes related to different phenotypes [18–20]. However, a phenotype is not only regulated by genes but is also regulated at multiple other levels, including at the metabolite level. While transcriptome sequencing can provide important information about gene expression levels, it is unable to shed light on real metabolite levels in organisms, making it hard to confirm the critical pathways responsible for regulating specific characters. Therefore using metabolomics—an analytical approach used to study metabolites and understand a biosystem’s physiological and biochemical status in relation to phenotype [21] —together with transcriptomics to study glycogen traits should prove to be a powerful method to understand glycogen metabolism in oysters. Recently, the integration of such large-scale datasets, including the transcriptome and the metabolome, has been applied successfully in other mollusks to study the genetic basis of environmental responses [22]. Gracey et al. have utilized the transcriptome and metabolome to characterize spontaneous metabolic cycles in *Mytilus californianus* under subtidal conditions [22]. In addition, Xu et al. have yielded insights into population-asynchronous ovary development in *Coilia nasus* using the same integrated application of transcriptomics and metabolomics [23]. However, to date, no study has investigated quality traits in oysters utilizing an integration of the transcriptome and the metabolome. Therefore, to promote our realization of the regulation of glycogen content, we performed an integration analysis including metabolites profiling, together with an analysis of transcript. To reveal the genetic mechanisms that determine oyster glycogen content, we selected the top 10–15 high and low glycogen content individuals from 185 half-sibling oyster families raised under a consistent environmental condition and used them to conduct transcriptome and metabolome analysis. Connection networks were mapped on the basis of correlations between metabolites and regulatory genes associated with glycogen content difference. The findings provide new insights into the study of the molecular mechanisms associated with nutritional traits and highlight the significance of an integrated approach for such research.

## Methods

### Experiment materials

Half-sibling families (thirty male oysters individually mated to an egg mix from thirty female oysters) were constructed and cultured on a farm in Jiaonan, Qingdao, China. No specific permissions were required for the present study and all experiments were conducted with approval from the Experimental Animal Ethics Committee, Institute of Oceanology, Chinese Academy of Sciences, China. At the age of sixteen months, 185 individuals were selected randomly and used for metabolome and transcriptome analysis. The adductor muscle was dissected from the oyster, and the remaining tissues were ground in the presence of liquid nitrogen and mixed together to form a powdered tissue extract which was then stored at −80 °C before analysis. This powdered tissue extract was then used for the measurement of glycogen content, as well as for transcriptome and metabolome analysis. Thirty oysters, out of the initial 185 individuals, consisting of fifteen individuals with the highest glycogen content, and fifteen individuals with the lowest glycogen content, were selected for transcriptome analysis. The top ten oysters with the highest glycogen content and the ten oysters with the lowest glycogen content, were used for metabolome analysis. Shell height, length, and width, total weight, and tissue weights were measured before dissection.

### Glycogen content assay

The glycogen content of the entire tissues without the adductor muscle, was detected using a kit for detecting liver and muscle glycogen content (Nanjing Jiancheng Bioengineering Institute, Nanjing, China). The procedure used was as follows: after tissues were ground into a powder in the presence of liquid nitrogen, a 0.50 μg sample was added to a tube containing alkaline liquor. The samples were incubated at 100 °C for 20 min in a water bath. After that, the hydrolysate was diluted 16-fold by the addition of distilled water. Subsequently, 2 mL of color reagent was added to the diluted hydrolysate and the samples were incubated for 5 min at a 100 °C in a water bath. Finally, the OD value of each sample was measured at 620 nm using a Multiscan Spectrum with a path length of 1 cm. Set the blank and standard for each group (Additional file 1: Table S1). The glycogen content was calculated according to the following formula: where 1.11 is the conversion coefficient of glucose content to glycogen content in this method. 0.01 is the glycogen content in the standard group. The coefficients 20 and 10 respectively represent the dilution ratio of the sample before and during detection.

$$ \mathrm{Glycogen}\  \mathrm{content}\ \left(\mathrm{mg}/\mathrm{g}\right)=\left(\frac{\mathrm{OD}\ \mathrm{of}\ {\mathrm{test}\  \mathrm{group}}^{\ast }}{\mathrm{OD}\ \mathrm{of}\  \mathrm{standard}\  \mathrm{group}}\right)\times 0.01\times 20\times 10\div 1.11 $$

* The OD value of each tube was measured at 620 nm wavelength.

### RNA sequencing data analysis

The same tissues used for glycogen content detection were also used for RNA extraction (i.e. the total tissue lacking the adductor muscle). To increase the accuracy, we increased the sample number in the transcriptome analysis to fifteen. RNA from the thirty oyster tissues (i.e. the fifteen high glycogen and fifteen low glycogen oysters) were exacted using TRIzol. Each of the thirty samples was used to generate an independent library. The thirty RNA-seq paired-end libraries were generated using NEBNext® Ultra™ RNA LibraryPrep Kit for Illumina® (NEB, USA) following the instruction’s procedure and index codes were added to attribute sequences to each sample. Briefly, poly-dT oligo-attached magnetic beads were used to extract mRNA from total RNA. mRNA fragmentation was performed under elevated temperature with divalent cations in NEBNext First Strand Synthesis Reaction Buffer. First strand cDNA synthesis was carried out using hexamer primers and M-MuLV reverse transcriptase (RNase H-) while the second strand was synthesis by DNA polymerase I and RNase H subsequently.

The remaining overhangs were converted into blunt ends using a combination of exonuclease/polymerase. To prepare for hybridization, NEBnext adapters with a hairpin loop structure were ligated to DNA fragments with the adenylated 3′ ends. After size selection (150 ~ 200 bp) of the ligation products, the ligated cDNA fragments that contained the adapter sequences were enhanced via PCR using PCR primers and Index (X) Primer. At the end, purification of PCR products were performed (AMPure XP system) and Agilent Bioanalyzer 2100 system were used for library quality assessment. Sequencing of libraries was performed using an Illumina Hiseq 2000 platform and 150 bp paired-end raw reads were generated. After removing adapter sequences, poly-N sequences, and low quality reads, clean reads were obtained. Meanwhile, the Q20, Q30, and GC content of the clean data were calculated.

### Read mapping and gene expression calculation

The HISAT2 v2.0.4 package [24] was used to map RNA-seq reads to the oyster genome (GenBank accession No., GCA_000297895.1) using default settings. The SAM files produced by HISAT2 were converted to BAM files and sorted using SAMtools v.1.3 [25]. The StringTie v1.3.3 package [26] was used to process the read alignments and the reference annotation with a parameter setting of “-eB”. Using this input, StringTie estimates abundance and creates a new transcript table for input to Ballgown. Gene expression levels were assessed using FPKM calculated using the R package Ballgown [27]. The pipeline for transcriptome analysis is shown in Additional file 2: Figure S1.

### Analysis of differentially expressed genes

Calculation of FPKM of each gene was performed by Cufflinks based on the length of gene read counts mapped to genome. Differential expression analysis was performed between high and low glycogen content groups using the DEGseq R package (1.10.1) [28]. Benjamini and Hochberg’s approach were used to control the false discovery rate (FDR) by resulting *P*-values adjustment. Genes with an adjusted *P*-value <0.001 found by DEGSeq were selected as differentially expressed. Principal component analysis (PCA) was performed using the SIMCA14 software package (Umetrics, Umea, Sweden).

Gene Ontology (GO) as an international standardized gene-function classification system consists of terms that provide a more global representation of gene functions using a controlled vocabulary. We selected 742 genes that had gene names annotated in the UniProt database from the 1888 differentially-expressed genes and transformed these gene names to protein names according to ID mapping on the UniProt website. GO enrichment was then analyzed using DAVID 6.8 Beta (david.ncifcrf.gov) and the results were classified with GOTERM_BP, GOTERM_CC, and GOTERM_MF. Significantly enriched metabolic and signal transduction pathways represented by DEGs were determined using pathway-enrichment analysis, compared with the whole genome background. KOBAS software were used to test the statistical enrichment of DEGs in KEGG pathways. A Q value ≤0.001 was considered as significantly enriched pathways among DEGs.

### Metabolite extraction and detection

0.05 g of each oyster tissue was extracted with 0.4 mL methanol–chloroform (3/1, *v*/v) and 20 μL of 2-Chloro-L-phenylalanine was added as an internal standard. The extract was homogenized using a ball mill for 4 min at 45 Hz, followed by centrifugation at 13,000 rpm for 15 min at 4 °C. Supernatant fluid (about 0.35 ml) was transferred to a new 2 ml glass vial. The extracts were dried under the vacuum concentrator without heating, and added 60 μL of methoxyamine hydrochloride (20 mg/mL in pyridine) to the dried metabolites, and then incubated for 30 min at 80 °C, after mixing and sealing. BSFTA reagent (80 μL in 1% TMCS, *v*/v) was then added to the samples and they were incubated for 2 h at 70 °C. Add FAMEs (5 μL), consisting of a standard mix of fatty acid methyl esters (C8–C16: 1 mg/mL; and C18–C24: 0.5 mg/mL in chloroform), to samples. Prior to GC–time-of-flight (TOF)–MS analysis, the samples were cooled to room temperature and fully mixed. GC-TOF/MS is a method that is capable of detecting low abundance metabolites, and has the ability to identify secondary metabolites as well as metabolic intermediates [29].

The detection of the metabolites were referred to methods of Hairui Wang et al. in 2017 [30]. In brief, GC–TOF–MS analysis was carried out utilizing an Agilent 7890 gas chromatograph system with the addition of a Pegasus 4D time-of-flight mass spectrometer. A DB-5MS capillary column were applied in this system. The column with 30 m × 250 μm inner diameter, 0.25 μm film thickness were coated with 5% diphenyl cross-linked with 95% dimethylpolysiloxane (J&W Scientific, Folsom, CA, USA). Splitless mode were used for injection with Helium as the carrier gas. The front inlet purge and gas flow rate were 3 mL/min and 1 mL/min respectively. The temperature program was 80 °C for 1 min, followed by raised to 290 °C at a rate of 10 °C per min and finally held at 290 °C for 5 min. Temperature for injection, ion source, and transfer line were 280, 220, and 295 °C respectively, with the −70 eV energy in electron impact mode. Mass spectrometry data were collected in full-scan mode with a range of 30–600 m/z at a rate of 20 spectra per second after a solvent delay of 7 min [30].

Chroma TOF 4.3X software from LECO Corporation and the LECO-Fiehn Rtx5 database were used for raw peak exacting, data baseline filtering and calibration of the baseline, peak alignment, deconvolution analysis, peak identification, and integration of the peak area. Peak identification were performed by retention time index method with the 5000 RI tolerance.

### Multivariate statistical analysis of metabolites

PCA (principal component analysis) and OPLS-DA (orthogonal projections to latent structures–discriminate analysis) were performed by SIMCA14 software package (Umetrics, Umea, Sweden) with the results of three-dimensional data including the peak numbers (which is a metabolite marker), sample names, and normalized peak areas. PCA was applied to distribute the original data while OPLS-DA were performed for obtaining of effective group separation and better understanding of factors responsible for the classification. The parameters of classification were R2Y = 0.961 and Q2Y = −0.113 which indicated adequate goodness-of-fit and predictive ability.

### Identification of significantly different metabolites and pathways between high and low glycogen content oysters

As described above, OPLS-DA was used to identify significantly different metabolites between the high- and low-glycogen content groups. To clarify this analysis, the VIP (variable importance for the projection) values along the predictive component were obtained. VIP values exceeding 1.0 were first selected as being changed metabolites. T test (Student’s t test) were used to the evaluation of remaining variables. Discard the variables with *P* > 0.05 between the two comparison groups. The fold change (FC) value of each metabolite was calculated by comparing the mean value of the peak area obtained from the high-glycogen content group to that from the low-glycogen content group. The metabolites were blasted against the metabolite database of *Caenorhabditis elegans,* which is evolutionarily close to *C. gigas.* Search the online databases involving the Kyoto Encyclopedia of Genes and Genomes (KEGG), and NIST (http://www.nist.gov/index.html) to further identify and confirm differential metabolites. Each differential metabolite and related pathways were cross-listed in KEGG. Additionally, the top enriched pathways were selected and finally constructed in accordance with the potential functional analysis.

### Integrative analysis of metabolome and transcriptome

Pearson correlation coefficients were calculated for metabolome and transcriptome data integration. MetScape 3 was utilized to visualize and clarfy the metabolomic and transcriptome data. This allow us to build networks of genes and metabolites. It also contribute to visualize metabolites difference and identify enriched pathways from transcriptome data.

## Results

### Metabolome analysis of the oysters with high glycogen and low glycogen

SPSS version 16.0 was used to build a descriptive statistical analysis of the glycogen content, as well as to construct a correlation analysis between glycogen content and shell height, shell length, shell width, total weight, and tissue weight. The glycogen content of the experimental oysters ranged from 3.1 to 35.7 mg/g, with a mean ± std. value of 16.3 ± 5.3. Glycogen content was normally distributed (*P* > 0.05), with more than ten-fold degree of variation. (Additional file 3: Figure S2). A correlation analysis showed that oyster glycogen content had a positive correlation with both total weight and tissue weight (*p* < 0.01) (Additional file 4: Table S2).

**Fig. 1:**
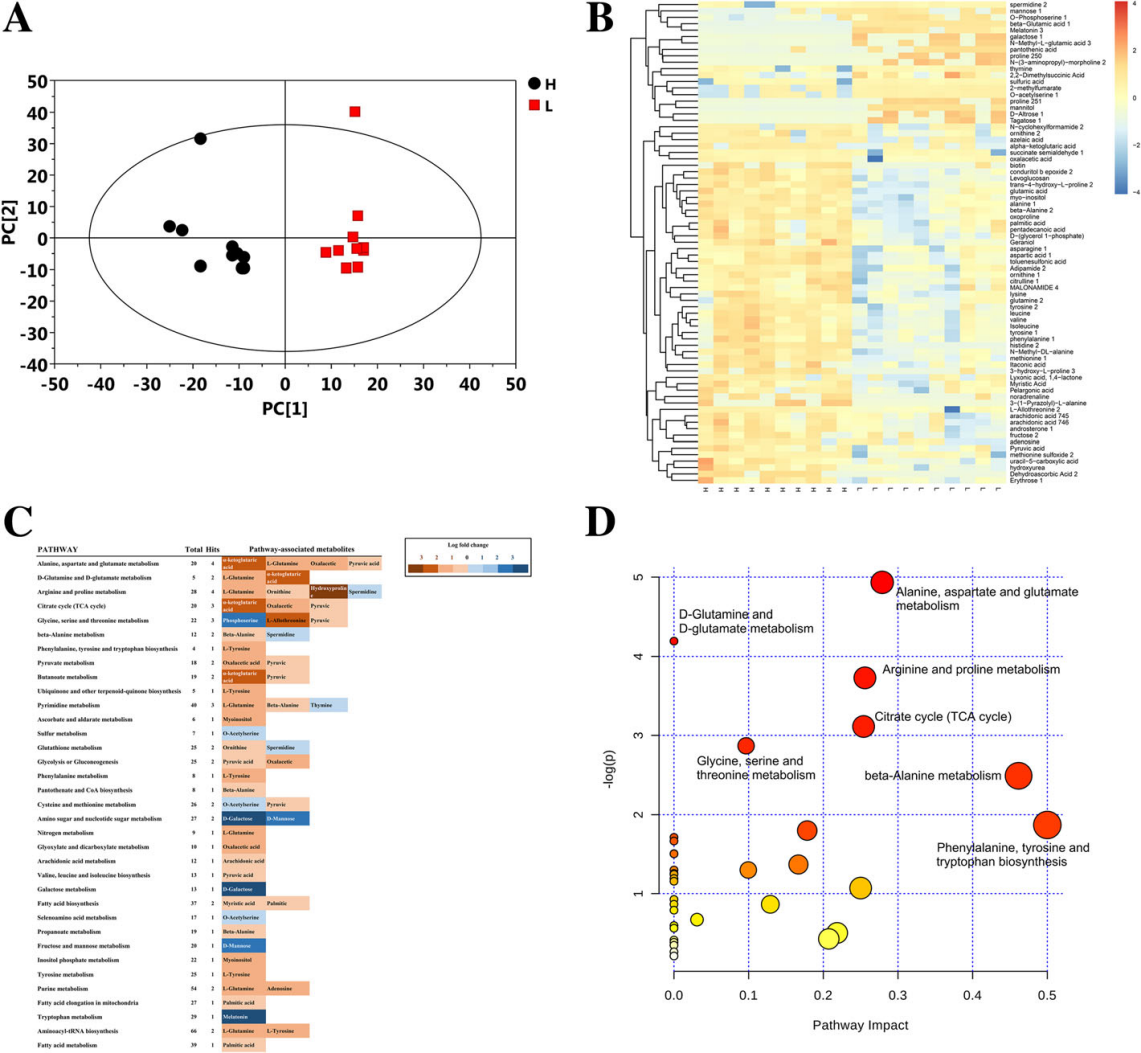
Metabolome analysis of oysters with high glycogen and low glycogen. **a** Metabolic profiles of high- and low-glycogen content oysters visualized by principal component analysis. The points represent the scores of biological replicates. **b** Heat map of all identifiable metabolites in Pacific oyster tissues comparing the high-glycogen group with the low-glycogen group. Columns represent biological replicates (*n* = 10 per group), and rows represent individual metabolites. The more abundant metabolites in the high-glycogen content oysters appear red, and the more abundant metabolites in the low-glycogen content oysters appear blue. The dendrograms denote the overall similarity of metabolite expression profiles (y-axis, left). **c** Pathway analysis for the identified metabolites in high- and low-glycogen content oysters. A row represent the enriched pathways and boxes correspond to difference in the relative abundance between high- and low-glycogen content oysters (metabolites more abundant in the high-glycogen content oysters appear red, and blue represents a more abundant metabolite in the low-glycogen content oysters). Total represent the total number of metabolites in the pathway and hits show the number of metabolites in the pathway significantly different between the two groups. **d** Statistical significance and pathway impact of the pathway analysis with MetaboAnalyst 3.0

Representative chromatograms of the oyster tissue samples analyzed by GC-MS are shown (Additional file 5: Figure S3). To compare the metabolite composition of the two oyster groups having either high- or low-glycogen content, both hierarchical cluster analysis (HCA) and principal component analysis (PCA) models were tested using the data obtained from GC-TOF-MS analysis. The percentage of explained value in the metabolome analysis of PC1 and PC2 was 13.2% and 9.5% respectively. Based on the PCA (Fig. 1a) and the heat map analysis (Fig. 1b), there was an obvious separation between the high- and low-glycogen content oysters.

**Fig. 2:**
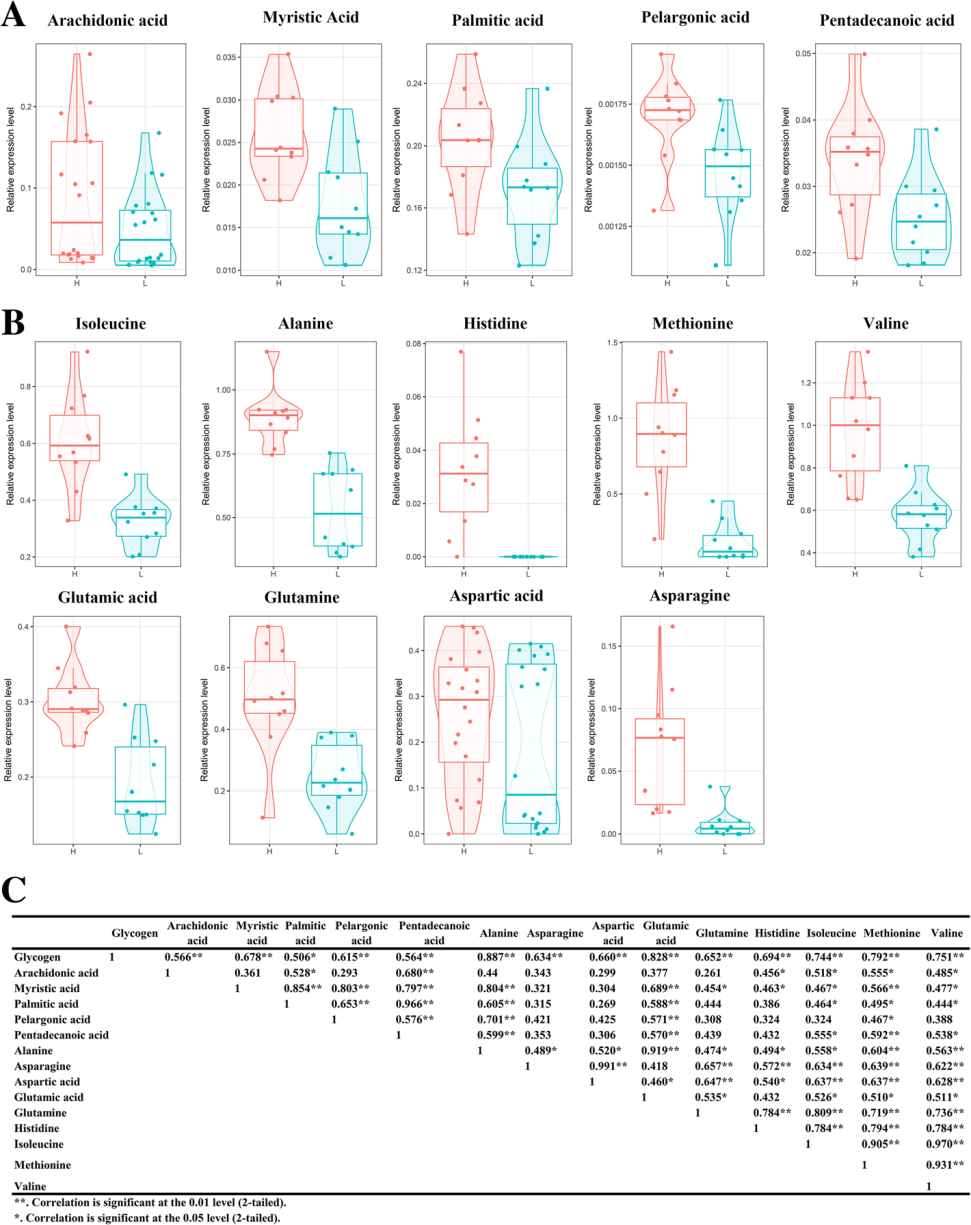
Amino acid and fatty acid contents and correlation with glycogen content. Significantly changed amino acids (**a**) and fatty acids (**b**) were detected by metabolomics analysis and were correlated with glycogen content (**c**). Metabolites in the high-glycogen oysters are shown in red, while blue represents metabolites in the low-glycogen oysters. **c** Correlation analysis between glycogen content and amino acid and fatty acid. ** Correlation is significant at the 0.01 level (two-tailed). * Correlation is significant at the 0.05 level (two-tailed)

In total, seventy-five differentially-abundant metabolites were identified from 767 peaks in the chromatograms, including amino acids, fatty acids, and sugars (Additional file 6: Table S3). In the high-glycogen content group, fifty-six metabolites were significantly up-regulated and nineteen were significantly down-regulated compared with the low-glycogen content group. Specific metabolite information and the concentrations in the high- and low- glycogen groups are shown in Additional file 7: Figure S4. The metabolome view map revealed that thirty pathways (Additional file 8: Table S4 and Fig. 1c) were enriched based on the seventy-five differentially-abundant metabolites originally identified between the two groups. However, only four of these thirty pathways displayed significant enrichment (P<0.05) namely, “alanine, aspartate and glutamate metabolism”(map00250), “d-glutamine and d-glutamate metabolism”(map00471), “arginine and proline metabolism”(map00330), and “citrate cycle (TCA cycle)”(map00020) (Fig. 1d). These enriched pathways were related to amino acid metabolism and glycogen metabolism. Interestingly, five fatty acids (palmitic acid, myristic acid, arachidonic acid, pelargonic acid, and pentadecanoic acid) and nine amino acids (alanine, aspartic acid, asparagine, glutamic acid, glutamine, histidine, methionine, isoleucine, and valine) were found to be abundant in high-glycogen content oysters (Fig. 2a, b). A correlation analysis conducted using SPSS version 16.0 showed that glycogen content had a positive correlation with the individual amino acid and free fatty acid levels (Fig. 2c). Besides this, pyruvic acid, oxaloacetic acid, and alpha-ketoglutaric acid, which are involved in the TCA cycle, were also found to be abundant in high-glycogen content oysters (Additional file 7: Figure S4).

### Transcriptome analysis of oysters with high-glycogen and low-glycogen contents

**Fig. 3:**
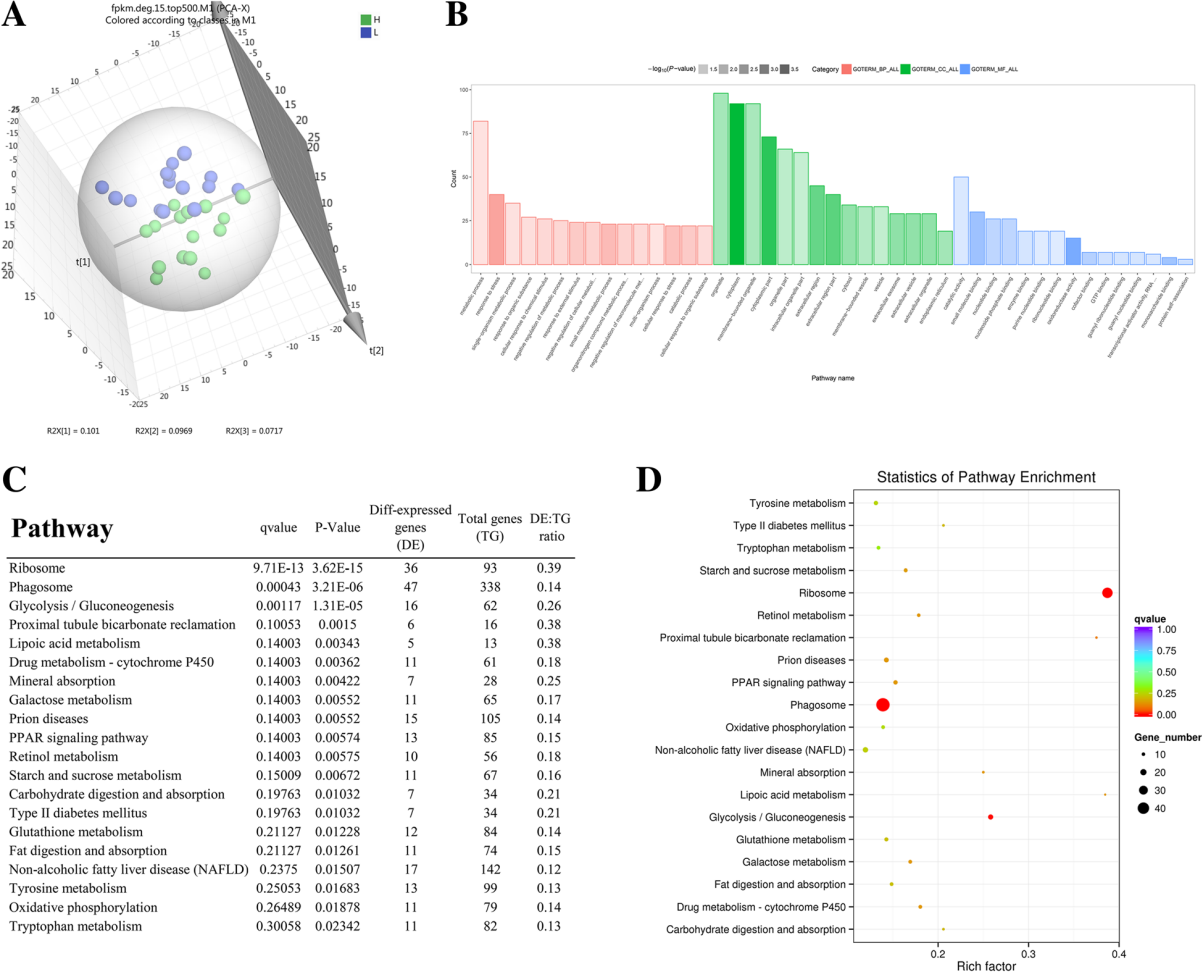
Transcriptome analysis of the oysters with high-glycogen and low-glycogen contents. **a** Principle component analysis of genes differentially expressed between the two groups. The points represent scores of biological replicates (15). **b** Gene Ontology (GO) terms enriched in genes differentially-expressed between the two groups. The bars denote the proportion of the differentially expressed genes relative to the total number of genes in the *C. gigas* genome mapped to each term. The depth of the color bars represents the *P* value of the term. The red, green, and blue bars represent the GOTERM_BP, GOTERM_CC and, GOTERM_MF categories respectively. **c** The top-twenty pathways enriched by genes differentially-expressed between high- and low-glycogen content oysters. DE: the number of genes in the pathway that were significantly differentially expressed between the two groups. TG: total number of genes in the enriched pathway. **d** Scatterplot of the KEGG pathway enriched by the differentially expressed genes. The vertical axis represents the name of the pathway and the horizontal axis shows the enrichment factor. The size of the plot denotes the number of differentially expressed genes while the color corresponds to the Qvalue. A deeper color represents a smaller Qvalue and indicates more significant enrichment of the pathway

cDNAs prepared from the thirty oysters representing the two group were sequenced individually using an Illumina Hiseq2000 platform. An average of 11.9 and 12.5 million high-quality 150 bp paired-end reads (107 GB reads in total) were mapped to the *C. gigas* genome from the fifteen high- and low-glycogen content oysters, respectively (Additional file 9: Table S5). A total of 25,911 genes had reads that mapped to the *C. gigas* genome and the average map rate was 73.55%. The transcriptomes of the two groups of oysters were compared using a principle component analysis (PCA), which allowed an unbiased analysis in a format in which the groups could be visually and quantitatively compared. The percentage of explained value of PC1, PC2, and PC3 in the transcriptome was 10.1%, 9.7%, and 7.2% respectively. Figure 3a shows that the high- and low-glycogen content oysters could be perfectly separated.

A total of 1888 differentially-expressed genes (DEGs) were identified between the two glycogen content groups (geometric algorithm, *P*-value <0.001). The expression level of the genes mentioned are shown in Additional file 10: Table S6. Compared to the low-glycogen content group, a total of 981 genes were up-regulated and 907 genes were down-regulated in the high-glycogen content group.

The DEGs were annotated according to Gene Ontology (GO) and KEGG. The majority (732) of the DEGs mapped to at least one GO term, and 181 GO terms were significantly enriched. Among these, “biological process”, “response to stress”, “single-organism metabolic process” and “negative regulation of metabolic process” were the most highly represented (Fig. 3b).

We used the method described by Kobas [31] to find pathways in the KEGG database that were significantly altered in the transcriptome. We mapped all DEGs to KEGG pathways and twenty-nine significant specific pathways (*P* < 0.05) were represented (Additional file 11: Table S7 and Fig. 3c). Among these twenty-nine pathways “Ribosome”, “Phagosome”, “Glycolysis/Gluconeogenesis” were the three most-represented pathways (Fig. 3c and d). Glycolysis and gluconeogenesis are both important pathways in glucose metabolism. The rate-limiting enzymes hexokinase (HK) and pyruvate kinase (PK) in glycolysis, and phosphoenolpyruvate carboxykinase (PEPCK) in gluconeogenesis, were highly expressed in high-glycogen oysters (Additional file 10: Table S6).

### Key pathways related to oyster glycogen content difference analysis by metabolome and transcriptome

**Fig. 4:**
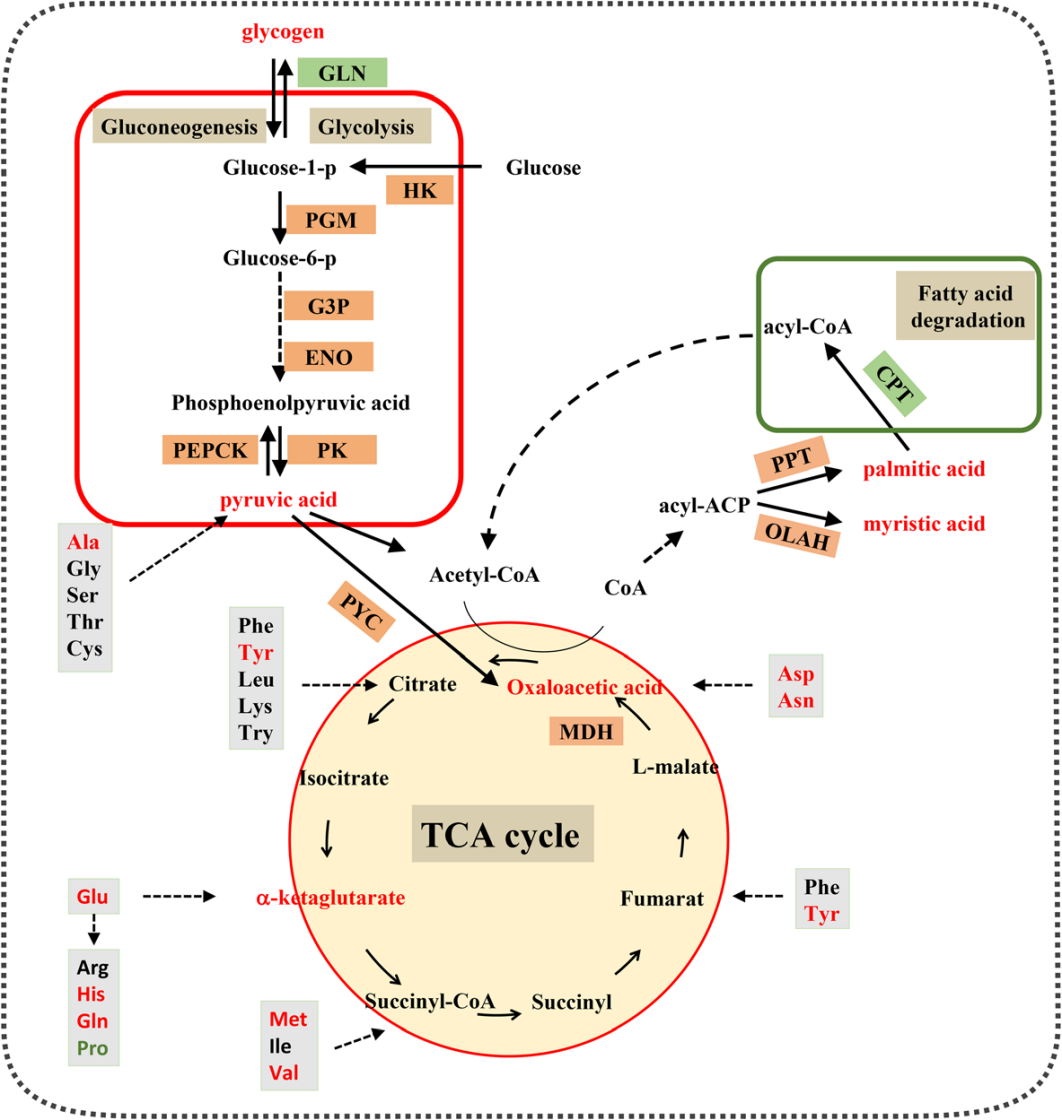
Pathway overview of different glycogen content enriched by metabolome and transcriptome in Pacific oysters. The differentially-expressed gene abbreviations identified by the transcriptome analysis are shown in either red boxes (highly expressed in high-glycogen oysters) or green boxes (highly expressed in low-glycogen oysters). Differentially-abundant metabolites identified by metabolome analysis are shown either in red lettering (highly expressed in high-glycogen oysters) or green lettering (highly expressed in low -glycogen oysters). Abbreviation: OLAH: oleoyl-[acyl-carrier-protein] hydrolase, PPT: palmitoyl-protein thioesterase, HXK: hexokinase, PGM: phosphoglucomutase, G3P: glyceraldehyde-3-phosphate dehydrogenase, ENO: enolase, PK: pyruvate kinase, PYC: pyruvate carboxylase, MDH: malate dehydrogenase, CPT2: carnitine O-palmitoyltransferase 2, PEPCK: phosphoenolpyruvate carboxykinase, GLN: glycogenin

MetScape 3 was utilized to visualize and clarfy the metabolomic and transcriptome data [32]. This allow us to build networks of genes and metabolites. It also contribute to visualize metabolites difference and identify enriched pathways from transcriptome data. An overview of the differentially expressed genes and metabolites found between high glycogen oysters and low glycogen oysters is shown in Fig. 4. The pathways enriched between the high-glycogen content group and the low-glycogen content group are shown in Additional file 12: Figure S5. Specific pathways, metabolites, and gene information are shown in Additional file 13: Table S8. A total of twenty-seven signaling pathways played important roles between the two groups, including thirty-nine key metabolites and seventeen genes known to be involved in “Glycolysis and Gluconeogenesis”, “the TCA cycle”, “fatty de novo acid biosynthesis and beta-oxidation”, and “amino acid metabolism”. For instance, in the TCA cycle, abundant levels of oxaloacetic acid, alpha-ketoglutaric acid, and pyruvic acid (Fig. 1c and Fig. 4) were detected in high-glycogen oysters together with high levels of malate dehydrogenase (MDH) and pyruvate carboxylase (PYC) (Additional file 10: Table S6).

**Fig. 5:**
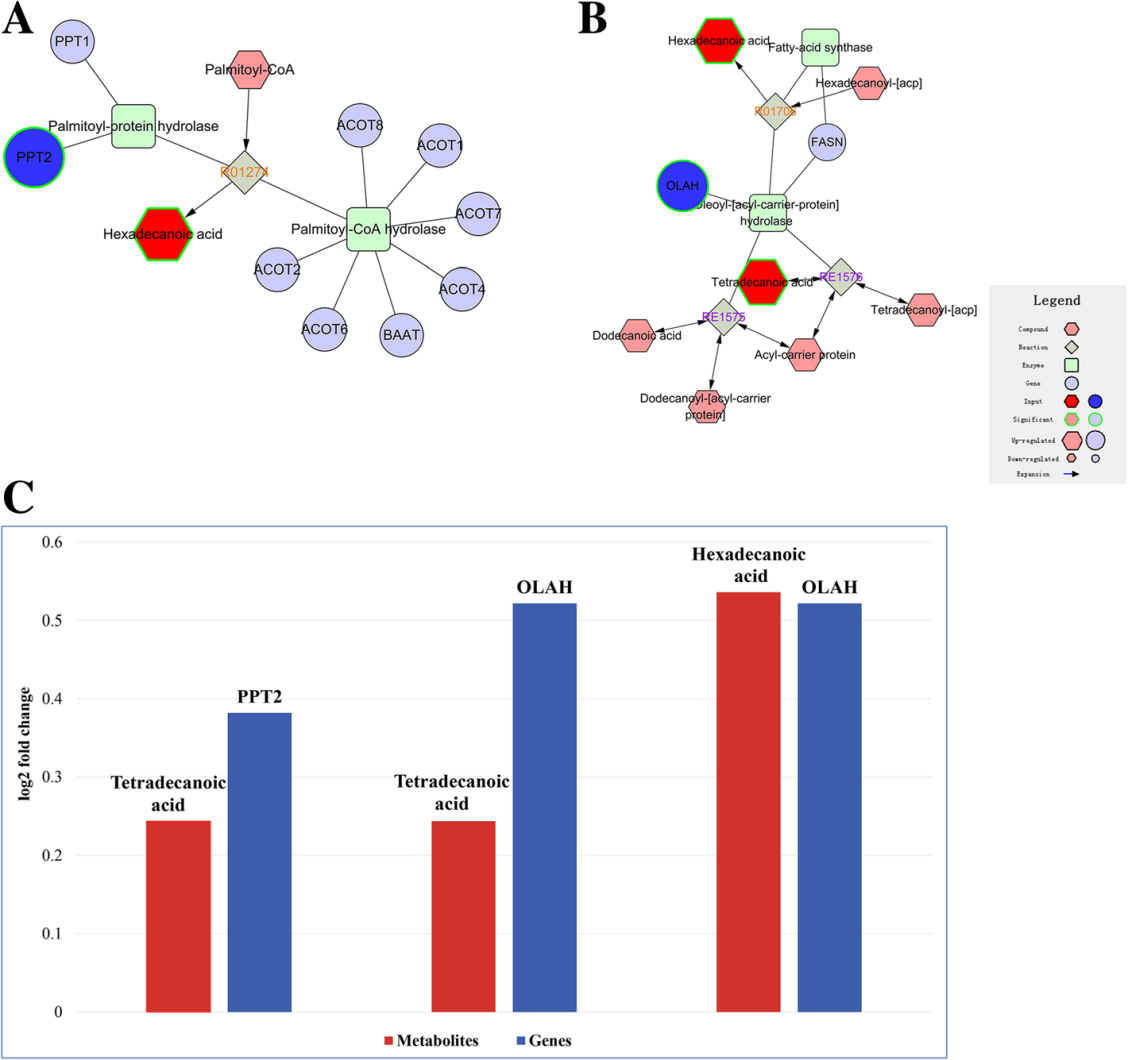
Enriched pathways include direct relation between gene expression levels and metabolite levels. **a** Saturated fatty acids and beta-oxidation. PPT2 (a differentially-expressed gene) releases hexadecanoic acid (a differentially-abundant metabolite) from hexadecanoyl-CoA according to reaction R01274. **b** De novo fatty acid biosynthesis. OLAH (a differentially-expressed gene) releases hexadecanoic acid and tetradecanoic acid (a differentially-abundant metabolite) from hexadecanoyl-acp and tetradecanoyl–acp according to reaction R01706. **c** Shows the levels of these differentially-expressed genes and differentially-abundant metabolites

In the above analysis, we found that three differentially-expressed genes and metabolites were directly related and were a cross validation (Fig. 5a and b). Oleoyl-ACP hydrolase (OLAH) and palmitoyl-protein thioesterase (PPT2) which are both hydrolases that participate in fatty acid biosynthesis are highly expressed in high-glycogen content oysters (Additional file 10: Table S6). Consistent with this, their products palmitic acid (hexadecanoic acid) and myristic acid (tetradecanoic acid) were also abundant in high-glycogen content oysters. PPT is a thioesterase that normally functions to remove long-chain fatty acids like palmitate from modified cysteine residues in proteins [33]. PPT releases hexadecanoic acid according to the R01274 reaction. Oleoyl-ACP hydrolase (OLAH) hydrolyzes acyl-ACP into the free fatty acid and ACP, thereby releasing the fatty acid from the fatty acid synthase complex [34]. OLAH releases hexadecanoic acid and tetradecanoic acid according to reactions R01706 and RE1576. The expression of these DEGs and the metabolites are shown in Fig. 5c.

Notably, three pathways that share a common enzyme, referred to as glutathione peroxidase 5 (GPX5), were identified based on an integrated analysis of both metabolome and transcriptome data (Additional file 14: Figure S6). These are the urea cycle, along with the metabolism of arginine, proline, glutamate, aspartate and asparagine, arachidonic acid metabolism, and linoleate metabolism. GPX5, which was highly expressed in high-glycogen content oysters, belongs to the glutathione peroxidase family and has the ability to reduce hydroperoxides [35].

## Discussion

Oyster glycogen content is a type of quantitative trait and varies extensively among different individuals [9, 36]. It is a complex and important trait that is affected by genetic and environment factors, including season, nutrients, salinity, and temperature [9, 37–39]. In this study, we created half-sibling families by individually mating thirty male oysters with an egg mix derived from thirty female oysters. The resulting oysters were raised under consistent environmental conditions such as temperature, salinity, and nutrient levels, in order to eliminate any potential environmental impact. One hundred and eighty-five *C. gigas* tissues obtained from the half-sibling families were used to determine glycogen content levels. We found there to be a significant range in glycogen content across all these samples. We then selected the top ten high-and low-glycogen contents individuals to conduct transcriptome and metabolome analysis. However, because of the significant variation among individuals, we increased the sample number in the transcriptome analysis to fifteen, which clearly provided an increase in accuracy compared to previous studies in which no more than five biological replicates were used [18, 22]. An integrative analysis of the transcriptome and metabolome allowed us to present a comprehensive overview between the genes and the metabolites controlling oyster glycogen content as shown in Fig. 4.

Based on the metabolome analysis, we identified seventy-five differentially-abundant metabolites. Among these seventy-five metabolites, nine glucogenic amino acids, and five free fatty acids were identified that had increased abundance in high-glycogen oysters and may therefore be related to regulating glycogen content in oysters. In addition, metabolites involved in the TCA cycle were also abundant in high-glycogen content oysters, which implied that oysters with higher glycogen content exhibited increased energy metabolism. Besides this, rate-limiting enzymes in glycolysis and the TCA cycle were also highly expressed in high-glycogen oysters, supporting this hypothesis.

### The glycogen content is close related to the fatty acid degradation capacity

There is experimental evidence to suggest that a high FFA concentration leads to the accumulation of glycogen through the suppression of glycolysis and TCA cycle [16, 40] but the molecular mechanism is not understood. Based on the data presented here, we hypothesize that the high glycogen content in oysters may be related to a decrease in expression of CPT2 gene and a decrease in the conversion of free fatty acids to acyl-CoAs. In our study, the “de novo fatty acid biosynthesis” pathway was highly expressed in high-glycogen content oysters along with abundant levels of myristic acid and palmitic acid. There was also high expression of OLAH and PPT, which participate in the release of fatty acids from the fatty acid synthase complex [33, 34]. In contrast to this, in low glycogen-content oysters, we showed that the rate-limiting enzyme of fatty acid degradation, namely carnitine O-palmitoyltransferase 2 (CPT2), was highly expressed and there was low expression of fatty acids like palmitic acid and myristic acid (Fig. 1c). CPT I and CPT II (Carnitine palmitoyltransferases I and II), together with the acyl-carnitine translocator, mediate the transfer of acyl-groups into mitochondria [41], and are the rate-limiting enzymes in fatty acid degradation and mitochondrial β-oxidation [42]. It has been reported in rats that a CPT inhibitor could inhibit FFA oxidation and thus affect glycogen levels [43]. This study is consistent with our result that FFA concentration was related to glycogen accumulation in the Pacific oyster. High levels of CPT expression are indicative of robust fatty acid degradation that would result in a decrease in the levels of free fatty acids along with an accumulation of acyl-CoA in low-glycogen oysters. This accumulation of long chain fatty acyl-CoA may inhibit glycogen synthase activity suppressing glycogen synthesis in low-glycogen oysters [44, 45]. In short, a high level of CPT2 expression increases flux through the fatty acid degradation pathway, causes a lowering of the free fatty acid levels and a reciprocal increase in long chain fatty acyl-CoA levels that then suppress glycogen accumulation.

### Amino acid content and conversion efficiency to glucose play crucial role in glycogen content

In our study, we observed an increase in the abundance of glucogenic amino acids, including alanine, aspartate, glutamic acid, as well as others, in high-glycogen oysters. There was also an increase in the level of pyruvic acid, as well as increased expression of PEPCK which catalyzes the first committed step in gluconeogenesis [46] in these same oysters. In rats, Nomura et al. found that Co^2+^-induced PEPCK suppression causes a reduction in glycogen content [47]. It has also been reported that in *Litopenaeus vannamei*, the gluconeogenic capacity of the hepatopancreas can be modulated by PEPCK activity which affects the glycogen content in shrimp [48]. It has also been reported that in humans, the glycogen content in liver was dependent on the activity of PEPCK [49]. These studies further validate our data whereby high levels of PEPCK expression correlate with high glycogen levels in the Pacific oyster. Taken together, we speculate that this increase in abundance of glucogenic amino acids (i.e. gluconeogenic substrates) coupled with an increase in gluconeogenic capacity leads to increased de novo production of glucose and ultimately to increased glycogen synthesis. In this model, the glucogenic amino acids are first transformed into the corresponding ketonic acid via a deamination reaction. Through the TCA cycle, these ketonic acids are transformed into pyruvic acid, which is then converted to oxaloacetate [50] and then to PEP by the action of PEPCK [46], leading to the generation of glucose. The high levels of PEPCK expression and the high pyruvic acid content imply a high level of gluconeogenesis in the high-glycogen content oysters. Together with the high levels of glucogenic amino acids, this will result in a high level of de novo glucose production leading to increased glycogen storage. A recent study that examined the effect of high protein feeding in rats also supported our hypothesis. Wistar rats fed a high protein diet also increased their glycogen content because a significant portion of the dietary amino acids was channeled to glycogen synthesis [17].

### Oysters with higher glycogen content exhibited increased energy metabolism

Based on an analysis of key metabolic pathway using the seventy-five differentially-abundant metabolites and the 1888 differentially-expressed genes, we observed that both the glycolytic pathway and the TCA cycle were enhanced in oysters with high-glycogen content, which implied increased energy metabolism occurs in the high-glycogen oysters (Fig. 4). HK and PK, which are both rate limiting enzymes in glycolysis and catalyze irreversible reactions, were found to be highly expressed in high-glycogen content oysters. Up-regulation of the TCA cycle was inferred by the increased abundance of oxaloacetic acid, alpha-ketoglutaric acid, and pyruvic acid, as well as increases in the expression of MDH and PYC. MDH and PYC play crucial roles in the citrate cycle wherein MDH catalyzes the interconversion of oxaloacetate and malate [51], while PYC catalyzes the formation of oxaloacetate from pyruvate with concomitant ATP cleavage [52]. Since glycogen is a glucose polysaccharide that serves as a form of energy storage [3], these data imply that there is an concurrent upregulation of the mobilization of glycogen through glycogen phosphorylase, with the resultant liberated glucose then completely metabolized to CO_2_ and H_2_O through glycolysis and oxidative metabolism (TCA cycle), along with the release of the energy.

The high level of expression of the HK and PK genes involved in glycolysis and the MDH and PYC genes that participate in the TCA cycle in high-glycogen oysters reveals that these oysters have a higher energy metabolism compared to low-glycogen content oysters and implies that they have a greater resistance to stress.

### Higher glycogen content oyster manifests as a higher anti-adversity ability

Interestingly, we also showed that the antioxidant enzyme GPX5 was highly expressed in the high-glycogen content oysters. The enzyme GPX5 was found to be in common with three pathways that were identified based on an integrated analysis of both the metabolome and the transcriptome. Glutathione peroxidase (GPX) is an antioxidant that has the ability to reduce hydroperoxides to alcohols and hydrogen peroxide to water in a glutathione-dependent reaction [35]. The high expression of GPX5 implies there is an increased antioxidant capacity, which is expected to contribute to an ability of the high-glycogen oysters to cope with stress. Glycogen may therefore play an important role in antioxidant defense and stress resistance. It has been reported that glycogen levels are massively reduced under hypo-osmotic stress [53], and that when the glycogen is exhausted, the osmotic imbalance caused by salinity stress is more severe [54]. It has also been reported that glycogen could be a potential biomarker for Cu-induced toxic effects in the Pacific oyster [55]. Although it has been widely recognized that glycogen participates in stress resistance in the oyster, the molecular mechanism behind this has not been identified. Making use of integrated analysis of transcriptome and metabolome, we suggest that GPX5 may be an important enzyme in the mechanism by which glycogen contributes to stress-resistance in oysters.

## Conclusion

In this study, we first generated the regulatory networks associated with differences in glycogen content using an integrated analysis of the metabolome and transcriptome in Pacific oysters with different intrinsic levels of glycogen. We have identified 1888 differentially-expressed candidate genes, and seventy-five differentially-abundant metabolites and conducted a correlation analysis between these differentially-abundant metabolites and these differentially-expressed genes. Our study provides new insights into the regulatory mechanism responsible for glycogen content differences in oysters. By combing these two approaches, we suggest that both amino acid and free fatty acids are closely related to glycogen content in oysters. Both a high fatty acid degradation capacity and a high glucogenic amino acid content contribute to the increased glycogen in high-glycogen content oysters. In addition to this, high-glycogen content oysters have a high-energy metabolism, as well as an increased antioxidant capacity, which contributes to their increased stress resistance. Our study has not only revealed the molecular basis of the relationship between amino acids, fatty acids, and glycogen, but also provides a molecular explanation for stress resistance in oysters. These findings provide new insights into the study of quality traits and could promote research into the molecular breeding of oysters.

## Additional files

Additional file 1: Table S1. Operation table for the glycogen content assay. (XLS 24 kb)

Additional file 2: Figure S1. Pipeline for transcriptome analysis. (PDF 195 kb)

Additional file 3: Figure S2. Histogram of glycogen content. The vertical axis represents the frequency and the horizontal axis shows the glycogen content. (PDF 658 kb)

Additional file 4: Table S2. Correlation analysis between glycogen content and growth traits. ‘*’ indicates the relationship between the characteristics reached a significant level (*P* < 0.05) and ‘**’ indicates the relationship between the characteristics reached an extremely significant level (*P* < 0.01). (XLS 25 kb)

Additional file 5: Figure S3. Gas chromatography coupled with mass spectrometry total ion chromatograms for the thirty samples. Red shows representative GC-TOF/MS ion chromatograms for the low-glycogen content group, whereas black shows representative GC-TOF/MS ion chromatograms for the high-glycogen content group. (PDF 582 kb)

Additional file 6: Table S3. Differentially-abundant metabolites identified by GC-TOF/MS. Data is presented as mean values ± SE (*n* = 10). Significant differences between the two groups were assessed by t test. Significant differences were declared at the level of *p* < 0.05 and VIP > 1. (H:high-glycogen content oysters, L: low-glycogen content oysters). (XLS 2133 kb)

Additional file 7: Figure S4. The expression level of metabolites detected by the metabolomics analysis. Differentially-abundant metabolites in the high-glycogen oysters are shown in red while blue represents differentially-abundant metabolites in low-glycogen oysters. (PDF 4652 kb)

Additional file 8: Table S4. KEGG pathway enrichment by metabolites. MetaboAnalyst3.0 (http://www.metaboanalyst.ca) was used for the pathway enrichment and topology analysis of the differentially-abundant metabolites. (XLS 33 kb)

Additional file 9: Table S5. Statistics of the transcriptome sequencing data. High: high-glycogen content oysters, Low: low-glycogen content oysters. (XLS 33 kb)

Additional file 10: Table S6. Expression levels and annotation of the genes referred to in this article. (XLS 36 kb)

Additional file 11: Table S7. KEGG pathways enriched from the transcriptome analysis. (XLS 30 kb)

Additional file 12: Figure S5. Co-regulation network of differentially-abundant metabolites and differentially-expressed genes. (PDF 1124 kb)

Additional file 13: Table S8. Metabolites and genes in enriched pathways identified by an integrative analysis of the metabolome and transcriptome data. The bold names represent the gene name whereas the others are metabolite names. (XLS 34 kb)

Additional file 14: Figure S6. Enriched pathways containing the GPX5 enzyme (A) Urea cycle and metabolism of arginine, proline, glutamate, aspartate, and asparagine (B) Linoleate metabolism and (C) Arachidonic acid metabolism. (PDF 434 kb)

## Abbreviations

CPT2: Carnitine O-palmitoyltransferase 2
DEGs: Differentially expressed gene
GC-TOF/MS: GC–time-of-flight (TOF)–MS
GO: Gene ontology
GPX5: Glutathione peroxidase enzyme 5
GWAS: Genome Wide Association Study
HK: Hexokinase
MDH: Malate dehydrogenase
mRNA: Messenger RNA
OLAH: Oleoyl-ACP hydrolase
OPLS–DA: Orthogonal projections latent structures–discriminate analysis
PCA: Principal component analysis
PEPCK: Phosphoenolpyruvate carboxykinase
PK: Pyruvate kinase
PPT2: Palmitoyl-protein thioesterase
PYC: Pyruvate carboxylase
QTL: Quantitative trait loci
VIP: Variable importance for the projection
