# Supplementary figures and images for: Integrated application of transcriptomics and metabolomics provides insights into glycogen content regulation in the Pacific oyster Crassostrea gigas
Source: BMC Genomics. 2017 Sep 11;18:713. doi: 10.1186/s12864-017-4069-8 (PMC5594505; doi:10.1186/s12864-017-4069-8)

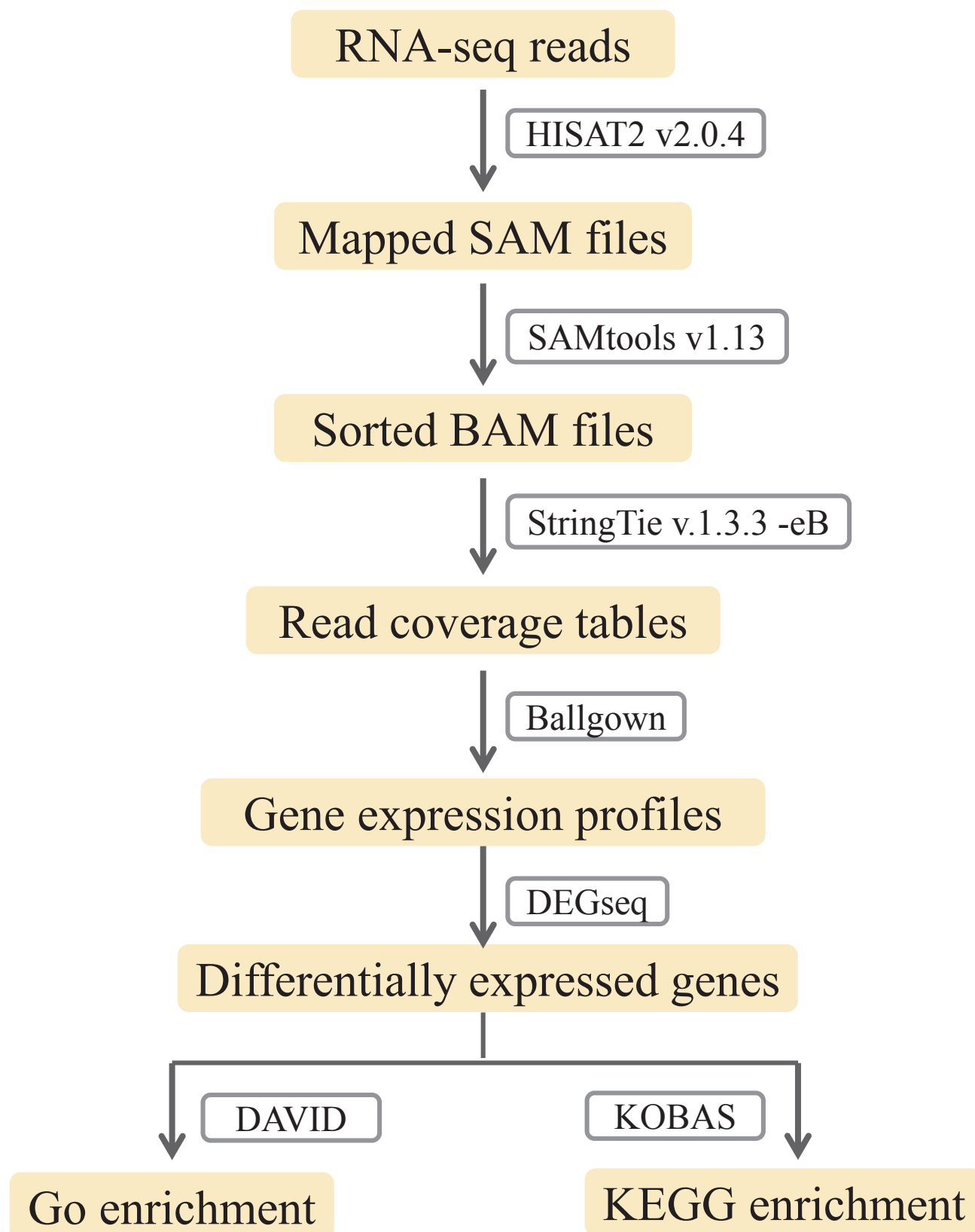

**Figure S1** Pipeline for transcriptome analysis.

Supplement: Supplementary file 2 — Pipeline for transcriptome analysis. (PDF 195 kb) [file 12864_2017_4069_MOESM2_ESM.pdf]
