# Supplementary material for: Integrated application of transcriptomics and metabolomics provides insights into glycogen content regulation in the Pacific oyster Crassostrea gigas
Source: BMC Genomics. 2017 Sep 11;18:713. doi: 10.1186/s12864-017-4069-8 (PMC5594505; doi:10.1186/s12864-017-4069-8)
Supplement: Supplementary file 3 — Histogram of glycogen content. The vertical axis represents the frequency and the horizontal axis shows the glycogen content. (PDF 658 kb) [file 12864_2017_4069_MOESM3_ESM.pdf]

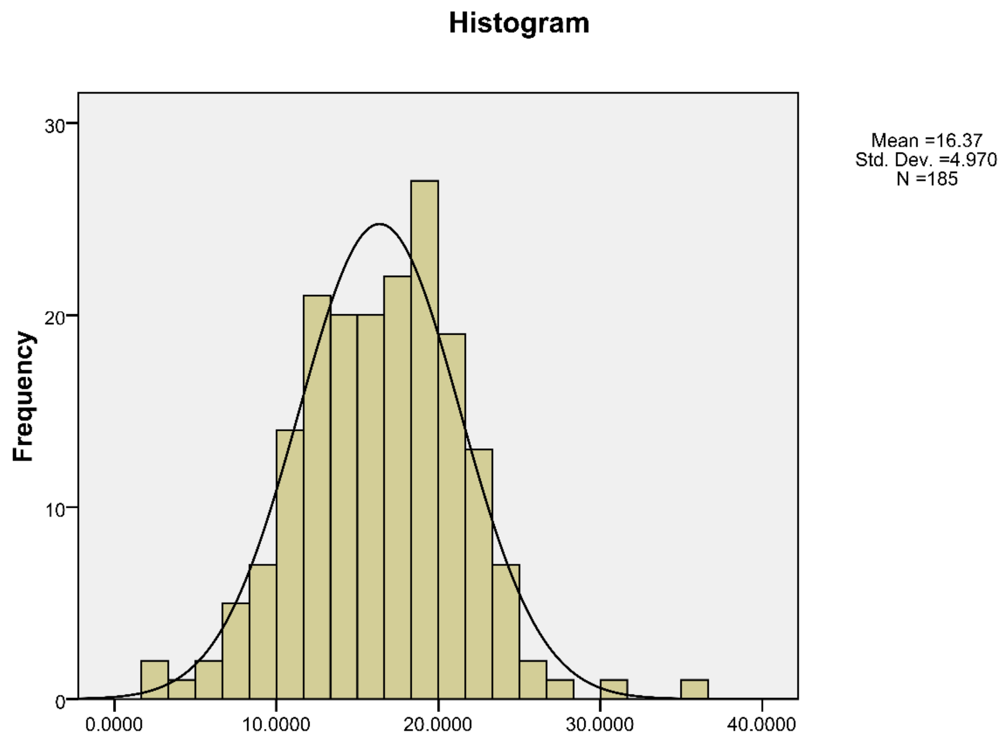

Figure S2 Histogram of glycogen content.The vertical axis represents the frequency and the horizontal axis shows the glycogen content.
