# Supplementary material for: Integrated application of transcriptomics and metabolomics provides insights into glycogen content regulation in the Pacific oyster Crassostrea gigas
Source: BMC Genomics. 2017 Sep 11;18:713. doi: 10.1186/s12864-017-4069-8 (PMC5594505; doi:10.1186/s12864-017-4069-8)
Supplement: Supplementary file 5 — Gas chromatography coupled with mass spectrometry total ion chromatograms for the thirty samples. Red shows representative GC-TOF/MS ion chromatograms for the low-glycogen content group, whereas black shows representative GC-TOF/MS ion chromatograms for the high-glycogen content group. (PDF 582 kb) [file 12864_2017_4069_MOESM5_ESM.pdf]

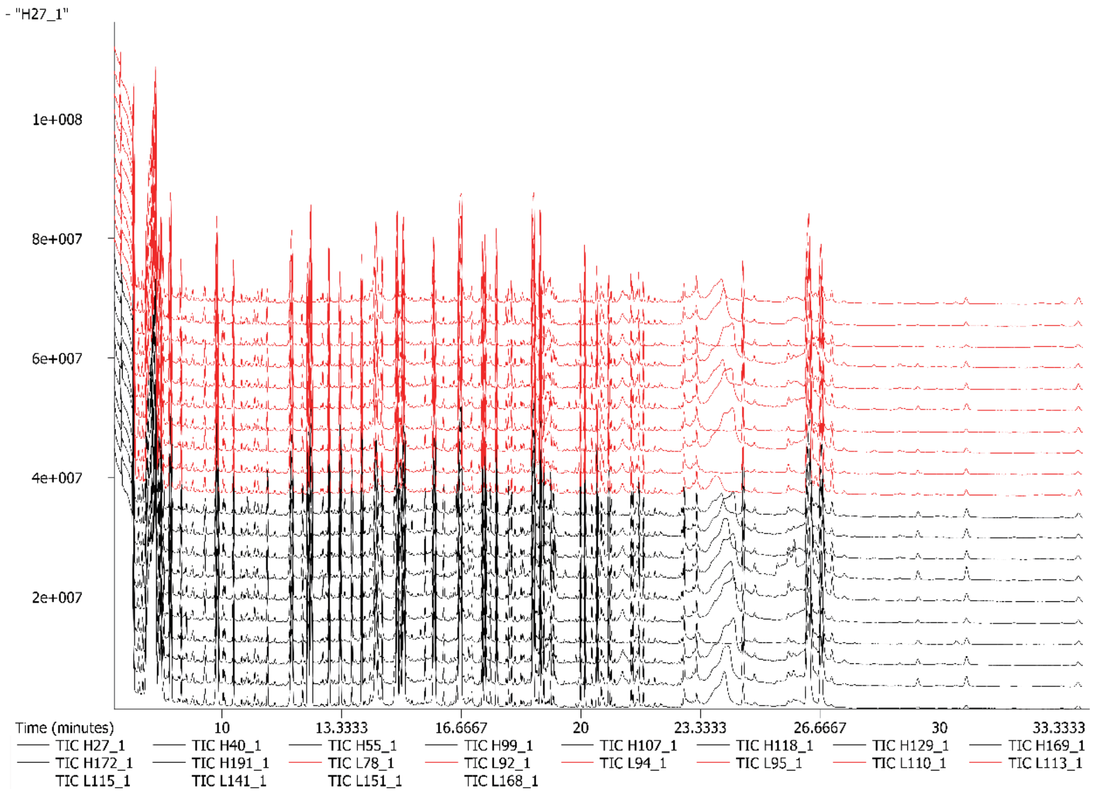

Figure S3 Gas chromatography coupled with mass spectrometry total ion chromatograms for the thirty samples. Red shows representative GC-TOF/MS ion chromatograms for the low-glycogen content group, whereas black shows representative GC-TOF/MS ion chromatograms for the high-glycogen content group.
