# Supplementary material for: Integrated application of transcriptomics and metabolomics provides insights into glycogen content regulation in the Pacific oyster Crassostrea gigas
Source: BMC Genomics. 2017 Sep 11;18:713. doi: 10.1186/s12864-017-4069-8 (PMC5594505; doi:10.1186/s12864-017-4069-8)
Supplement: Supplementary file 7 — The expression level of metabolites detected by the metabolomics analysis. Differentially-abundant metabolites in the high-glycogen oysters are shown in red while blue represents differentially-abundant metabolites in low-glycogen oysters. (PDF 4652 kb) [file 12864_2017_4069_MOESM7_ESM.pdf]

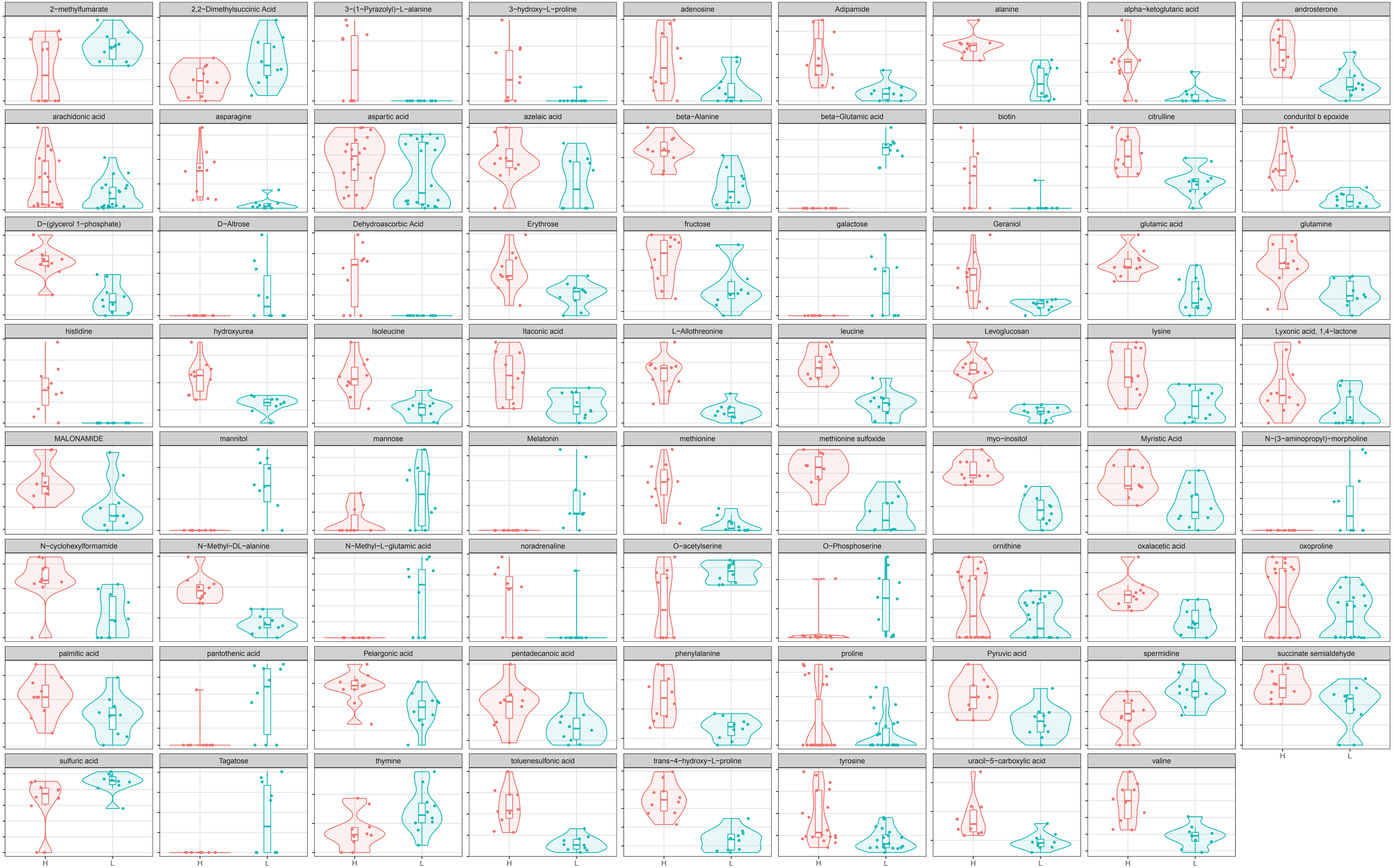

Figure S4 The expression level of metabolites detected by the metabolomics analysis. Differentially-abundant metabolites in the high-glycogen oysters are shown in red while blue represents differentially-abundant metabolites in low-glycogen oysters.
