# Supplementary material for: Integrated application of transcriptomics and metabolomics provides insights into glycogen content regulation in the Pacific oyster Crassostrea gigas
Source: BMC Genomics. 2017 Sep 11;18:713. doi: 10.1186/s12864-017-4069-8 (PMC5594505; doi:10.1186/s12864-017-4069-8)
Supplement: Supplementary file 14 — Enriched pathways containing the GPX5 enzyme (A) Urea cycle and metabolism of arginine, proline, glutamate, aspartate, and asparagine (B) Linoleate metabolism and (C) Arachidonic acid metabolism. (PDF 434 kb) [file 12864_2017_4069_MOESM14_ESM.pdf]

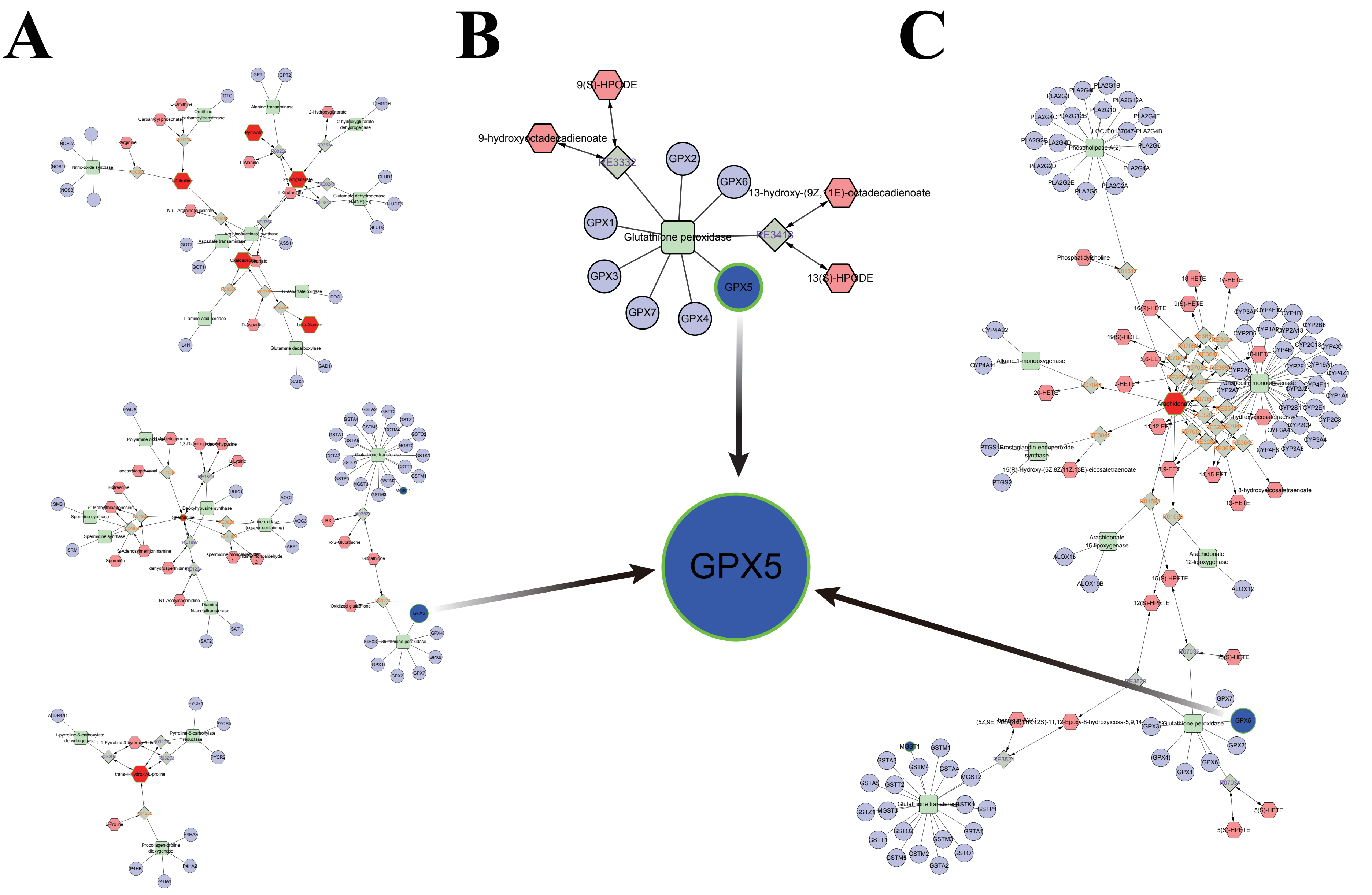

Fig S6 Enriched pathways containing the GPX5 enzyme (A) Urea cycle and metabolism of arginine, proline, glutamate, aspartate and asparagine (B) Linoleate metabolism and (C) Arachidonic acid metabolism .
